# Beverage Co-Consumption Profiles, Sleep, and Fatigue in Professional Army Personnel

**DOI:** 10.3390/nu18172930

**Published:** 2026-09-07

**Authors:** Vicente Javier Clemente-Suárez, Jan Florian, Rodrigo Yáñez-Sepúlveda, Julio A. Ceniza-Villacastín, Jose Francisco Tornero-Aguilera, Alexandra Martín-Rodríguez

**Affiliations:** 1 Department of Sport Sciences, Faculty of Sport and Health Sciences, Fit Generation Research Institute, AD400 Andorra la Vella, Andorra; jtornero@fitgeneration.es; 2 Grupo de Investigación en Cultura, Educación y Sociedad, Universidad de la Costa, Barranquilla 080002, Colombia; 3 University of Defence, 66210 Brno, Czech Republic; jan.florian@unob.cz; 4 Faculty of Education and Humanities, School of Sports Science, Universidad Andrés Bello, Viña del Mar 2520000, Chile; rodrigo.yanez.s@unab.cl; 5 School of Medicine, Universidad Espíritu Santo, Samborondón 091650, Ecuador; 6 Strength Training and Neuromuscular Performance Research Group (STreNgthP_RG), Faculty of Health Sciences HM Hospitals, University Camilo José Cela, 28692 Madrid, Spain; julioalfonso.ceniza@alumno.ucjc.edu; 7 Grupo de Pesquisa Atleta Tático (GPAT), Brazilian Army, Rio de Janeiro 22291-090, Brazil; 8 Faculty of Health Sciences, UNIE University, 28015 Madrid, Spain; sandra.martin.rodriguez8@gmail.com

**Keywords:** military nutrition, Spanish Army, caffeine, energy drinks, sugar-sweetened beverages, sleep, fatigue, latent class analysis

## Abstract

**Background/Objectives:** Active-duty military personnel may use caffeinated and sweetened beverages to manage alertness and fatigue, but co-occurring beverage behaviours are rarely examined. We identified beverage-frequency co-consumption profiles in Spanish Army personnel and tested their cross-sectional associations with self-reported sleep and habitual physical fatigue. **Methods:** This cross-sectional study included 1155 active-duty Spanish Army personnel. Latent classes were estimated from ordinal frequencies of caffeinated beverages, sugar-sweetened beverages, and energy drinks; these indicators represented frequency categories rather than caffeine dose, sugar intake, or standardized serving size. Adjusted outcome models incorporated classification uncertainty and controlled for demographic, occupational, training, hydration, alcohol, smoking, and recruitment-cohort covariates. **Results:** A three-class solution identified lower-frequency (47.7%), intermediate-frequency (28.3%), and higher-frequency (24.0%) profiles. Compared with the lower-frequency profile, the higher-frequency profile was associated with 0.230 h less sleep per night (95% CI −0.416 to −0.045; q = 0.022), 2.129 percentage points lower sleep efficiency (95% CI −4.177 to −0.082; q = 0.041), and 0.395 points greater habitual physical fatigue (95% CI 0.077 to 0.714; q = 0.022). Sensitivity analyses were directionally consistent. **Conclusions:** Beverage-frequency responses clustered into three profiles. The higher-frequency profile was cross-sectionally associated with shorter self-reported sleep, lower sleep efficiency, and higher habitual physical fatigue. The sleep-duration contrast was approximately 13.8 min/night, but the clinical or perceptible relevance of the sleep-efficiency and fatigue differences cannot be established from these self-reported measures. Prospective studies with product-specific dose and timing data are required to establish temporality and practical relevance.

## 1. Introduction

Nutrition is a determinant of military readiness because it supports sustained physical work, cognitive vigilance, recovery, and long-term health. Contemporary military nutrition frameworks therefore treat food and beverage provision as a capability that spans preparation, performance, and recovery rather than as a peripheral wellness issue [1,2]. A recent systematic review of available defence samples reported poor-to-fair diet quality, including inadequate intake of nutrient-dense foods and substantial exposure to discretionary products [3]. Interventions that modify the military food environment can improve food selection, but most initiatives have concentrated on meals and general diet quality rather than on the beverages used to manage alertness and fatigue [4].

Military service creates a setting in which stimulant use is both understandable and potentially self-reinforcing. Night duties, early starts, irregular shifts, deployment demands, extended training, and restricted sleep opportunities encourage personnel to use caffeine to preserve vigilance and task performance. Caffeinated products are widely used across military populations, and younger personnel appear particularly likely to consume energy drinks [5,6,7,8,9]. Caffeine can improve alertness, reaction time, endurance, and selected occupational tasks, especially under sleep loss [10,11]. These short-term benefits, however, must be balanced against the possibility that poorly timed or high-dose intake reduces subsequent sleep and perpetuates reliance on stimulation during the following duty period.

The sleep effects of caffeine are supported by convergent experimental evidence. A systematic review and meta-analysis estimated that caffeine reduced total sleep time by approximately 45 min and sleep efficiency by about seven percentage points, while increasing sleep-onset latency and wakefulness after sleep onset [12]. A subsequent randomized crossover trial demonstrated that the effect depends strongly on dose and timing: 100 mg taken four hours before bedtime produced little measurable disruption, whereas 400 mg impaired sleep even when consumed substantially earlier [13]. A 2025 systematic review in athletes similarly concluded that caffeine used to enhance performance may compromise subsequent sleep and recovery [14]. Recent wearable and self-report data in healthy young adults have reinforced the need to distinguish product, dose, habitual use, and timing [15]. Interindividual sensitivity also varies; differences in caffeine metabolism and adenosine-receptor signalling, including variation involving CYP1A2 and ADORA2A, have been discussed as contributors to heterogeneous responses [16]. These distinctions are directly relevant to military personnel, whose intake may occur during extended duties rather than at standardized times.

Energy drinks complicate this relationship because they combine caffeine with sugar or non-nutritive sweeteners and, frequently, taurine, B vitamins, botanical extracts, or other bioactive ingredients. Their caffeine concentration and serving size vary substantially, and their marketing emphasizes energy, performance, and concentration [16,17]. Military studies have associated frequent energy-drink consumption with short sleep, sleep disruption, fatigue, aggression, and psychological symptoms [8,18,19,20]. In a large national sample of university students, even occasional energy-drink consumption was associated with less sleep and poorer sleep characteristics, with progressively worse outcomes at higher frequencies [21]. Systematic reviews have also identified insomnia, jitteriness, and cardiovascular symptoms among the adverse outcomes reported after energy-drink use [22,23]. Although these findings do not establish causality, they show that energy drinks cannot be treated as nutritionally neutral alertness aids.

Sugar-sweetened beverages add a further dimension. Observational and review evidence links shorter sleep with more frequent intake of sugar-sweetened beverages, but directionality and mechanism remain uncertain [24,25]. Sleep restriction may alter appetite regulation, reward sensitivity, and beverage choice, whereas frequent intake of sweetened beverages may also occur within dietary, social, or occupational patterns that independently influence sleep. These associations should not be generalized to carbohydrate-containing foods as a whole, because food matrix, macronutrient composition, dose, and timing differ materially from sugar-sweetened beverage exposure [25]. Energy drinks may also be sugar-sweetened or non-nutritively sweetened, so energy-drink frequency cannot be interpreted as a measure of sugar exposure. Consequently, analyses that isolate one beverage while ignoring the wider pattern may miss co-occurring beverage behaviours relevant to military routines.

Dietary-pattern research addresses this limitation by examining combinations of intake rather than single products [26]. The three indicators were combined because the study aimed to characterize co-occurring beverage-frequency responses rather than construct a nutrient-dose score: the general caffeine item captured broad caffeinated-beverage frequency, the energy-drink item isolated a specific product category that may also contain caffeine and sugar, and the sugar-sweetened beverage item captured a broader sweetened-beverage behaviour. Latent class analysis is useful when indicators are categorical and have different response scales because it identifies unobserved subgroups with similar response profiles and estimates each participant’s probability of belonging to each subgroup [27,28,29,30]. The resulting classes are therefore interpreted as beverage-frequency response profiles, not as equivalent levels of caffeine dose, sugar intake, or standardized servings.

Despite extensive literature on caffeine and energy drinks, three gaps remain. First, most military studies have examined individual products or total caffeine rather than co-consumption configurations. Second, much of the cited military evidence derives from non-Spanish service populations [5,6,7,8,9,18,19,20], leaving limited evidence from Spanish Army personnel. Third, sleep duration, sleep efficiency, and physical fatigue have rarely been examined together as recovery-related correlates of beverage-frequency profiles after accounting for occupational workload, training, smoking, alcohol, hydration, and military rank. The present study therefore aimed to identify latent patterns of caffeinated-beverage, sugar-sweetened beverage, and energy-drink frequency in active-duty Spanish Army personnel and to test whether the higher-frequency profile was associated with shorter sleep, lower sleep efficiency, and greater physical fatigue. We hypothesized a graded pattern in which the higher-frequency class would show the least favourable recovery profile, while recognizing that the cross-sectional design could not establish whether beverage use preceded or followed sleep loss and fatigue.

## 2. Materials and Methods

### 2.1. Study Design and Participants

This cross-sectional secondary analysis used a cleaned database of 1155 active-duty Spanish Army personnel from two recruitment cohorts (1043 and 112 participants). Questionnaire responses were collected in Spain between 9 February and 19 May 2026 using self-administered Spanish-language Google Forms. The two questionnaires shared the demographic, psychological, sleep, nutrition, beverage, and work-environment core used in this analysis, although their physical-activity modules differed. Participants received standardized written introductory information describing the study as an evaluation of stress and modulating factors, participation as voluntary and anonymous, an estimated completion time of approximately 10 min, and the approved ethics and data-protection procedures. The analytical archive does not contain a recruitment log or standardized variable documenting the invitation pathway; therefore, the exact recruitment mechanism and participation rate cannot be reconstructed. Military activity at the moment of questionnaire completion (for example, routine duty, field exercise, guard duty, or post-exercise recovery) was not recorded.

Eligible participants were aged 18 years or older, had verified active-duty Spanish Army status, provided informed consent, and completed all three beverage-frequency indicators required for latent class estimation. The report follows the STROBE recommendations for cross-sectional studies [31]. The study was conducted in accordance with the Declaration of Helsinki and approved by the University Ethics Committee (approval code 2024-863; 14 October 2024). All participants provided informed consent before completing the questionnaire. Personal identifiers were removed before analysis. The complete respondent-facing questionnaires for the two Spanish Army recruitment cohorts are provided as Supplementary Materials.

### 2.2. Beverage-Frequency Indicators

Three study-specific ordinal questionnaire items were used exclusively as indicators in the latent class models. They were administered in Spanish and were not taken from a validated beverage-frequency questionnaire. The caffeinated-beverage question asked, “How often do you consume caffeinated beverages (coffee, tea, energy drinks)?” and was coded as 0 = never, 1 = 1–2 times per day, 2 = 3–4 times per day, and 3 = 5 or more times per day. The sugar-sweetened beverage question referred to soft drinks and packaged juices and was coded as 0 = rarely or never, 1 = 1–2 times per week, 2 = 3–4 times per week, and 3 = almost every day. The energy-drink question was coded as 0 = never, 1 = occasionally or for special events, 2 = 1–2 times per week, and 3 = 3 or more times per week.

The response intervals were pragmatic categories defined in the parent questionnaires; they were not derived from validated dose thresholds, and no formal cognitive interviewing or psychometric validation of these three beverage items was documented. No standardized serving size, product brand, caffeine concentration, sugar content, or time of consumption was collected. Thus, “1–2 per day” denotes a frequency response and not one or two standardized servings. The general caffeinated-beverage item explicitly listed energy drinks, creating partial conceptual overlap with the specific energy-drink indicator. Both were retained because the first represented broad caffeinated-beverage frequency and the second isolated energy-drink frequency as a product-specific behaviour. This overlap was considered in interpretation, while local-independence diagnostics and separate-indicator sensitivity models were used to assess whether the selected latent structure depended on a single indicator. Because category intervals differed between items, the scores were modelled as categorical labels and must not be interpreted as equivalent servings, caffeine doses, or amounts of added sugar.

### 2.3. Sleep and Fatigue Outcomes

Sleep questions referred to the previous month. Sleep duration was obtained from the item asking how many hours of actual sleep the participant had obtained per night on average. Time in bed was derived from the reported usual bedtime and wake time, and sleep efficiency was calculated as reported sleep duration divided by time in bed and multiplied by 100. Sleep-efficiency values were retained only when the underlying bedtime, wake-time, time-in-bed, and sleep-duration responses were internally consistent; values outside the plausible range were excluded before modelling. These questions correspond to domains assessed by the Pittsburgh Sleep Quality Index (PSQI) [32], but the complete PSQI was not administered and no PSQI global score was calculated.

Habitual physical fatigue was assessed only in the larger of the two Spanish Army recruitment questionnaires (n = 1043) using the question, “On a scale from 0 to 10, how would you rate your habitual level of physical fatigue?”, with higher scores indicating greater fatigue. The item assessed habitual rather than momentary fatigue, and questionnaire completion was not standardized to a specific time of day. Single-item fatigue measures have shown convergent validity in occupational research [33], but the exact habitual 0–10 item used here has not been independently validated in Spanish Army personnel. It was retained as a pragmatic low-burden self-report outcome in a questionnaire designed for approximately 10 min completion and was not interpreted as a clinical fatigue diagnosis. Sleep duration, sleep efficiency, and physical fatigue were analyzed as continuous outcomes because the purpose was to quantify mean differences between beverage-frequency profiles rather than define clinical categories.

### 2.4. Covariates

The primary adjusted models included age, sex, body mass index, military-rank group, weekly work hours, estimated weekly training volume, perceived excessive workload, recruitment cohort, water-intake score, alcohol-intake score, and smoking status. Age, sex, weight, and height were self-reported, and body mass index was calculated from weight and height. Military rank was grouped according to the Spanish Army hierarchy: troops comprised Soldado, Cabo, Cabo primero, and Cabo mayor; non-commissioned officers comprised Sargento, Sargento primero, Brigada, Subteniente, and Suboficial mayor; and officers comprised Alférez, Teniente, Capitán, Comandante, Teniente coronel, Coronel, and General. Weekly work hours were reported as average hours per week, including guards and maneuvers. Perceived excessive workload referred to the previous month and was coded from 0 = never to 4 = always.

Water intake was coded ordinally as 0 = fewer than 4 glasses/day (<1 L), 1 = 4–6 glasses/day (approximately 1–1.5 L), 2 = 7–8 glasses/day (approximately 1.5–2 L), and 3 = more than 8 glasses/day (>2 L). Alcohol intake was coded as 0 = never, 1 = occasionally (1–2 times/month), 2 = weekly (1–2 times/week), and 3 = frequently (3 or more times/week). Smoking status was represented by daily, occasional, former, and never smoking categories. Estimated weekly training volume was harmonized across the two questionnaires: in the larger questionnaire it was calculated as weekly running minutes plus weekly strength-training minutes; in the smaller questionnaire it was calculated as reported sessions per week multiplied by the midpoint of the selected duration category (22.5, 45, 75, or 105 min). The recruitment cohort was therefore retained as an adjustment variable to account for questionnaire-module differences.

Covariates were selected before outcome modelling from variables available in the parent dataset that could plausibly relate to beverage selection, sleep, or fatigue on the basis of the military caffeine and energy-drink literature [5,6,7,8,9,18,19,20]. Combat, support, and administrative duty categories were not collected, so military rank should not be interpreted as a substitute for occupational role. Unit fixed effects were added in a sensitivity analysis to account for stable differences between participating units. Additional sensitivity-only covariates included a single-item previous-month sleep-quality rating, perceived stress measured with the 10-item Perceived Stress Scale (PSS-10) [34], and depressive symptoms measured with the Patient Health Questionnaire-9 (PHQ-9) [35]. These measures were administered in Spanish; no independent psychometric revalidation was undertaken specifically in this Spanish Army sample.

### 2.5. Latent Class Analysis

The objective of latent class analysis was to identify unobserved subgroups of participants with similar response patterns across the three beverage-frequency indicators. Using maximum likelihood, the algorithm estimated each participant’s probability of belonging to each candidate class and iteratively updated class parameters to improve fit to the observed response distribution [27,28,29,30]. Locally independent models containing one to five classes were fitted to the three four-category indicators using an expectation–maximization algorithm. The indicators were modelled as multinomial rather than assuming equal distances between response categories. Candidate models were estimated from 20 random sets of starting values, and the selected solution was re-estimated from 100 random starts to assess convergence to the same maximum.

Model enumeration prioritized the Bayesian information criterion (BIC), with additional consideration of the Akaike information criterion (AIC), consistent AIC (CAIC), sample-size-adjusted BIC (SABIC), entropy, smallest modal class size, interpretability, stability across starts, and local-independence diagnostics [27,28,29,30]. Lower AIC, BIC, CAIC, and SABIC values indicate better relative fit after their respective complexity penalties; entropy values closer to 1 indicate clearer probabilistic separation of classes. Class size, substantive interpretability, convergence across random starts, and local-independence diagnostics were considered alongside information criteria rather than using any single index mechanically. The selected three-class solution was labelled lower-frequency, intermediate-frequency, and higher-frequency according to its conditional response probabilities. These labels describe relative frequency-response patterns within this sample and are not diagnostic or dose-based categories.

### 2.6. Outcome Models and Sensitivity Analyses

Associations between latent class and each outcome were estimated using multivariable linear regression with HC3 heteroskedasticity-robust variances. Classification uncertainty was propagated using posterior pseudo-class multiple imputation: class membership was drawn from each participant’s posterior class probabilities in 300 datasets, the adjusted model was fitted in each dataset, and estimates and variances were combined using Rubin’s rules [29,36]. The lower-frequency class was the reference. Higher-versus-lower contrasts constituted the prespecified primary family; intermediate-versus-lower contrasts were secondary. The Benjamini–Hochberg procedure controlled the false discovery rate across the three primary outcomes [37]. Two-sided *p* values and 95% confidence intervals are reported, with q < 0.05 interpreted as evidence after multiplicity correction.

Sensitivity analyses tested whether the findings depended on class categorization or unit structure. These analyses included: (i) an ordered class score representing a one-class increase; (ii) modal class assignment with unit fixed effects; (iii) a standardized continuous co-consumption score calculated as the mean of the three standardized beverage indicators; (iv) additional adjustment of the fatigue model for the previous-month sleep-quality item, PSS-10 perceived-stress score, and PHQ-9 depressive-symptom score; (v) mutually adjusted models containing the three beverage indicators separately; and (vi) repetition of model enumeration in the larger recruitment cohort. Local independence was examined using bivariate residuals for each indicator pair. Analyses were conducted in Python 3.11 using NumPy (https://numpy.org/, accessed on 5 September 2026) and SciPy (https://scipy.org/, accessed on 5 September 2026); reproducible code is provided as Supplementary Materials.

## 3. Results

The sample comprised 1155 active-duty Spanish Army personnel, including 1070 men and 85 women. Mean age was 29.15 ± 8.25 years and mean body mass index was 25.22 ± 2.67 kg/m^2^. Mean sleep duration was 6.12 ± 1.13 h. Sleep duration was available for 1153 participants before model-specific covariate restriction, sleep efficiency for 1018, and physical fatigue for 1043. Complete-case adjusted analyses included 1128 participants for sleep duration, 998 for sleep efficiency, and 1022 for physical fatigue.

The three-class model had the lowest BIC (8066.3), compared with 8075.0 for the two-class model and 8115.3 for the four-class model (Table 1). The four-class solution had a slightly lower AIC but a higher BIC and produced a smaller additional class without a substantively distinct response pattern. The selected model had entropy of 0.749 and a smallest modal class of 21.2%. When re-estimated from 100 random starts, 45 solutions converged within 0.01 log-likelihood units of the best solution. Bivariate residual tests did not provide evidence of substantial local dependence between indicator pairs (all *p* ≥ 0.098).

**Figure 1:**
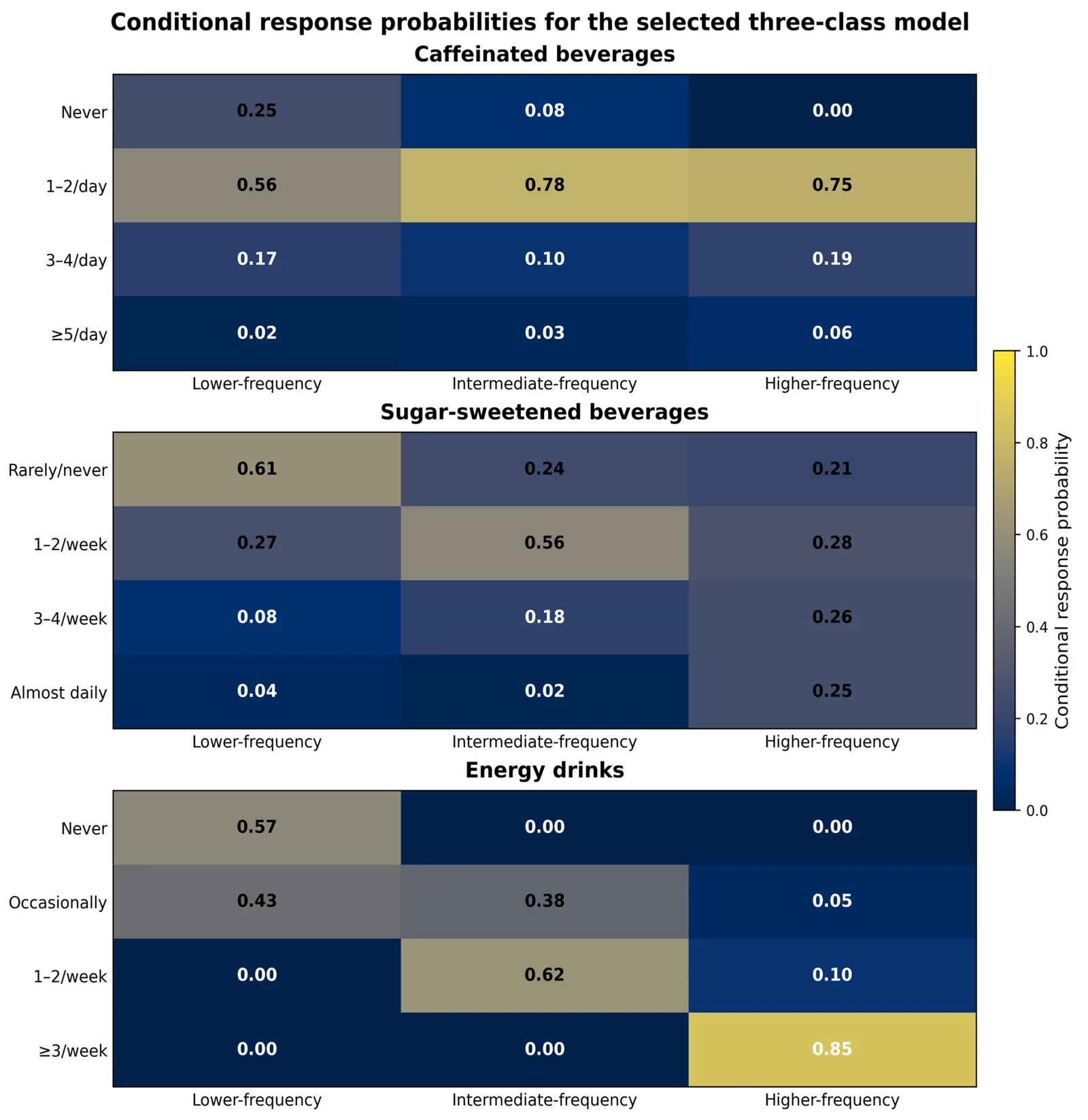
Conditional response probabilities for the selected three-class beverage-frequency model. Separate panels show caffeinated beverages, sugar-sweetened beverages, and energy drinks to improve readability. Values are estimated response probabilities conditional on latent-class membership. The general caffeinated-beverage item explicitly included energy drinks, so the first and third indicators are conceptually overlapping. Category intervals differ between indicators and must not be interpreted as equivalent servings, caffeine dose, sugar intake, or independent nutrient exposures.

Estimated class prevalences were 47.7% for the lower-frequency profile, 28.3% for the intermediate-frequency profile, and 24.0% for the higher-frequency profile. The lower-frequency profile was characterized by infrequent energy-drink and sugar-sweetened beverage responses; the intermediate-frequency profile was distinguished mainly by energy-drink use 1–2 times/week; and the higher-frequency profile by an 84.9% probability of energy-drink use at least three times/week together with more frequent sugar-sweetened beverage and caffeinated-beverage responses (Figure 1). Average posterior probabilities were 0.896, 0.788, and 0.988, respectively.

Modal summaries indicated that, compared with the lower-frequency profile, the higher-frequency profile was younger, contained a greater proportion of troops, reported more weekly training, and included more daily smokers (Table 2). Unadjusted sleep, sleep-efficiency, and fatigue values are presented in Table 2. These comparisons are descriptive because modal assignment ignores posterior uncertainty and the classes were not randomized.

**Figure 2:**
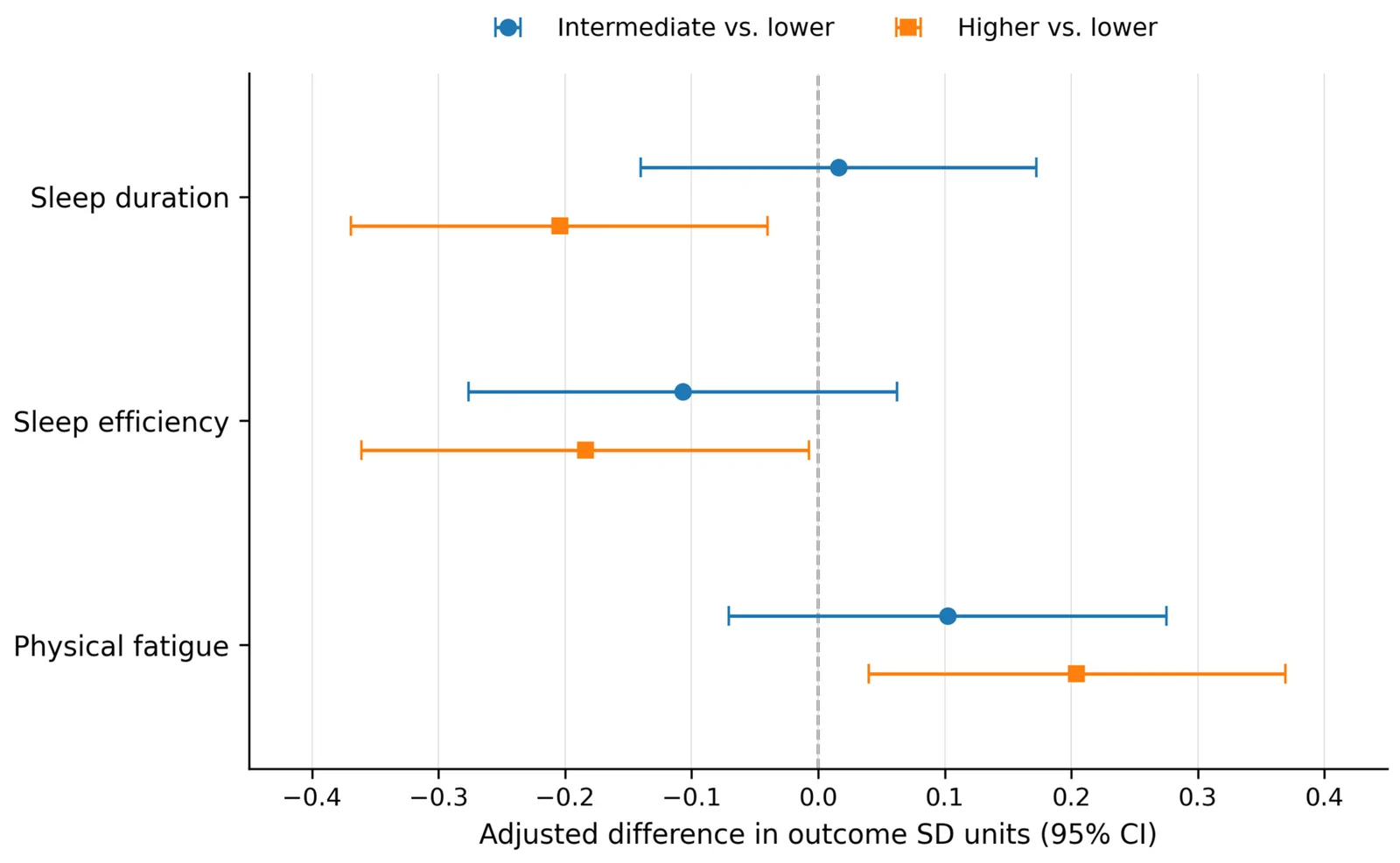
Adjusted class differences in standardized outcome units. Points show adjusted mean differences and horizontal lines show 95% confidence intervals. Standardization used the observed outcome standard deviation to permit comparison across sleep duration, sleep efficiency, and physical fatigue. Negative values indicate shorter sleep or lower efficiency; positive values indicate greater fatigue.

After covariate adjustment and propagation of class uncertainty, the higher-frequency profile reported 0.230 h less sleep per night than the lower-frequency profile (95% CI −0.416 to −0.045; *p* = 0.015; q = 0.022), equivalent to approximately 13.8 min. Sleep efficiency was 2.129 percentage points lower (95% CI −4.177 to −0.082; *p* = 0.041; q = 0.041), and habitual physical fatigue was 0.395 points higher on the 0–10 scale (95% CI 0.077 to 0.714; *p* = 0.015; q = 0.022). Intermediate-versus-lower contrasts were not statistically significant for sleep duration, sleep efficiency, or fatigue (Table 3; Figure 2).

Sensitivity analyses were directionally consistent with the primary models. Each one-class increase was associated with 0.106 h shorter sleep, 1.078 percentage points lower sleep efficiency, and 0.198 points greater fatigue; all three trends remained significant after false-discovery-rate correction. Modal-class models with unit fixed effects produced similar associations. A one-standard-deviation increase in the continuous co-consumption score was associated with 0.111 h shorter sleep, 0.902 percentage points lower sleep efficiency, and 0.198 points greater fatigue. The fatigue association remained after additional adjustment for the previous-month sleep-quality item, PSS-10 perceived stress, and PHQ-9 depressive symptoms. In contrast, when caffeinated beverages, sugar-sweetened beverages, and energy drinks were entered simultaneously as separate standardized predictors, no individual coefficient reached *p* < 0.05. Full sensitivity results are presented in Tables S3–S6.

## 4. Discussion

This study identified three recurring beverage-frequency response profiles in active-duty Spanish Army personnel. Compared with the lower-frequency profile, the higher-frequency profile was cross-sectionally associated with approximately 13.8 min shorter self-reported sleep per night, 2.129 percentage points lower derived sleep efficiency, and 0.395 points higher habitual physical fatigue on a 0–10 scale. These estimates describe differences between frequency-response profiles; they do not quantify caffeine or sugar dose, establish a clinically perceptible difference, or identify a causal effect. The intermediate-frequency profile did not differ significantly from the lower-frequency profile, and the main pattern remained directionally consistent across alternative representations of co-consumption.

The class structure is substantively plausible as a description of beverage-frequency behaviour, but not as a caffeine-dose gradient. The lower-frequency profile combined a high probability of never using energy drinks with infrequent sugar-sweetened beverage intake, whereas the higher-frequency profile had an 84.9% probability of consuming energy drinks at least three times per week. Caffeinated-beverage responses also differed across profiles, although less sharply: one to two caffeinated-beverage occasions per day remained the most probable category in all three profiles. Because the general caffeine question explicitly included energy drinks, some respondents’ energy-drink use may have contributed to both indicators. In addition, serving size, caffeine concentration, sugar content, and timing were not measured. The profiles should therefore be interpreted as overlapping beverage-frequency response patterns rather than levels of total caffeine or sugar exposure [16,17,23].

The demographic composition of the profiles provides additional context. In this sample, personnel in the higher-frequency profile were younger, were more often troops than those in the lower-frequency profile, reported more training, and were more likely to smoke daily. Younger age has also been associated with greater energy-drink use in previous military studies [5,6,7,8,9]. These descriptive differences do not explain the adjusted associations completely because age, rank, training, smoking, work hours, workload, alcohol, hydration, and recruitment cohort were included in the models. Nevertheless, residual differences in duty role, scheduling, peer norms, access to beverages, and other unmeasured characteristics remain plausible.

The adjusted sleep-duration difference between the higher- and lower-frequency profiles was 0.230 h, or approximately 13.8 min per night. The sample as a whole reported an average of 6.12 h/night, indicating that restricted sleep was common at group level; however, the present study did not prespecify or validate a minimal clinically important difference for self-reported sleep duration. The 13.8 min contrast should therefore not be labelled clinically meaningful or clinically negligible. It is substantially smaller than the average acute reduction reported in experimental caffeine studies [12], but direct comparison is limited because the present exposure was frequency-based, mixed across products, and assessed under habitual conditions. The design also permits reverse causation: personnel sleeping less may consume caffeinated or energy drinks more frequently to maintain alertness.

The adjusted difference in sleep efficiency was 2.129 percentage points. No validated minimal clinically important difference was available for the derived self-reported sleep-efficiency measure used here, and the questionnaire did not administer the complete PSQI or obtain actigraphy or polysomnography. The difference should therefore be interpreted as a statistical association rather than a diagnostic or individually perceptible change. Experimental evidence indicates that caffeine-related sleep disruption depends strongly on timing, dose, habitual exposure, and individual sensitivity [12,13,14,15,16]. The present frequency questions could not distinguish 100 mg consumed early in the day from a substantially larger dose consumed near bedtime, and they cannot identify whether the association reflects beverage exposure, reverse causation, or both.

The physical-fatigue difference requires similar restraint. The adjusted contrast of 0.395 points on a 0–10 habitual-fatigue scale was statistically significant, but no validated minimal clinically important difference exists for this exact item in Spanish Army personnel. Single-item fatigue measures can provide useful low-burden information in occupational settings [33], yet their interpretation depends on wording, reference period, and population. Caffeine is commonly used to counter sleepiness and perceived effort [10,11], so higher fatigue may have preceded and motivated more frequent beverage use. Conversely, frequent stimulant use may coexist with restricted recovery opportunities. The cross-sectional data cannot distinguish these temporal pathways, even after adjustment for measured sleep quality, perceived stress, and depressive symptoms.

The pattern-based analysis adds information beyond mutually adjusted individual-beverage models, but the result should be interpreted methodologically rather than biologically. None of the separate standardized indicators reached conventional significance when entered simultaneously, whereas latent and continuous co-consumption representations were associated with the outcomes. This does not demonstrate synergy among caffeine, sugar-sweetened beverages, and energy drinks. The indicators were ordinal, correlated, and partly overlapping because the general caffeinated-beverage item included energy drinks; these features can attenuate or redistribute separate coefficients. The non-significant local-dependence diagnostics and sensitivity analyses support the stability of the selected statistical pattern, but they do not convert the indicators into independent nutrient exposures. The more defensible interpretation is that person-centred and composite approaches summarize the beverage-frequency behaviour represented in this dataset [26].

The results extend previous military evidence while remaining specific to this dataset. Earlier deployment studies linked high energy-drink use with very short sleep and sleep disruption [18,19,20], and a focused military review concluded that higher caffeine intake was generally associated with shorter and less restorative sleep [5]. Recent studies in non-military young adults have reported similar frequency gradients for energy drinks and sleep [21], while systematic evidence links short sleep with greater sugar-sweetened beverage intake [24]. The present study adds a large Spanish Army sample, models several beverage-frequency indicators simultaneously, and carries classification uncertainty into adjusted analyses. It identifies a recurring co-consumption response profile for prospective evaluation rather than establishing a harmful beverage combination.

### 4.1. Practical Applications

The findings support targeted assessment rather than punitive restrictions. Because the higher-frequency profile was younger and reported more weekly training, particular attention may be warranted when young, physically active Spanish Army personnel report frequent energy-drink use together with short sleep or persistent fatigue. Screening should record beverage type, serving size, product-specific caffeine content, timing, purpose of use, sleep opportunity, and hydration rather than infer exposure from a single frequency item. A question about broad “caffeine” frequency is insufficient because coffee, tea, and energy drinks can differ markedly in caffeine dose and other ingredients.

Education can distinguish strategic caffeine use from habitual compensation for inadequate sleep and can reinforce routine hydration practices during training and duty. Ready access to water and unsweetened beverages, planned drinking opportunities, and attention to fluid intake are reasonable components of military health practice, particularly during periods of high physical activity. Guidance can also emphasize avoidance of unnecessarily high caffeine doses late in the duty cycle and awareness of caffeine from multiple products [1,2,3,4,16]. These recommendations are general risk-management considerations; the present cross-sectional study did not test a hydration intervention, a caffeine-reduction strategy, or a harmful consumption threshold.

### 4.2. Strengths and Limitations

Strengths include the large active-duty sample, the use of a person-centred model suited to categorical indicators, explicit evaluation of one- to five-class solutions, stability testing across random starts, local-independence diagnostics, propagation of posterior classification uncertainty, correction for multiple outcomes, and several sensitivity analyses. The study also separated the current hypothesis from previous analyses of the parent database that focused on military rank, broad dietary-pattern proxies, or body-mass-index and training profiles.

The limitations are substantial. First, the three beverage indicators were study-specific frequency questions rather than a validated beverage-frequency instrument. They did not measure product brand, standardized serving size, caffeine dose, sugar quantity, formulation, timing, reason for use, or co-ingestion with alcohol or supplements, and their response intervals were not equivalent across indicators. The broad caffeinated-beverage item explicitly included energy drinks, creating partial conceptual overlap with the energy-drink indicator. No formal cognitive interviewing or psychometric validation of these beverage items was documented. The latent classes therefore represent frequency-response patterns only and should not be interpreted as caffeine-dose or sugar-intake phenotypes.

Second, sleep and fatigue were self-reported. The sleep questions covered selected PSQI domains but did not constitute the complete validated PSQI, sleep efficiency was derived rather than objectively measured, and the habitual physical-fatigue item was a single study-specific 0–10 rating administered only in the larger recruitment questionnaire. The fatigue item was not independently validated in Spanish Army personnel, and survey completion was not standardized by time of day. Third, the exact recruitment pathway and participation rate could not be reconstructed from the analytical archive. The specific military activity being performed at questionnaire completion and combat/support/administrative occupational role were not recorded, leaving potential residual confounding by duty context.

Fourth, weekly training volume was harmonized from different physical-activity modules in the two recruitment questionnaires: running plus strength-training minutes in the larger questionnaire and sessions multiplied by a duration-category midpoint in the smaller questionnaire. The recruitment cohort was included in adjusted models, but measurement heterogeneity may remain. Missing outcome and covariate data reduced model-specific sample sizes, and complete-case modelling assumes that inclusion did not depend on unobserved values after accounting for measured variables. Fifth, the cross-sectional design prevents temporal or causal inference and leaves open the possibility that short or inefficient sleep and fatigue increased beverage use. Residual confounding by shift timing, chronotype, medications, supplements, health status, acute workload, and unit culture is likely. Finally, the sample was predominantly male and drawn from the Spanish Army, limiting generalizability to women, other service branches, reserve personnel, and non-military populations.

## 5. Conclusions

Caffeinated-beverage, sugar-sweetened beverage, and energy-drink frequencies formed three distinguishable co-consumption response profiles in active-duty Spanish Army personnel. The higher-frequency profile, representing approximately one quarter of the sample, was cross-sectionally associated with shorter self-reported sleep, lower derived sleep efficiency, and higher habitual physical fatigue than the lower-frequency profile. These classes are frequency-response patterns rather than estimates of caffeine dose or sugar intake, and the clinical or perceptible importance of the observed between-profile differences cannot be determined from the present measures. Because temporal order was not established, the findings do not support causal inference. Prospective studies should quantify product-specific caffeine and sugar exposure, record timing and purpose of use and concurrent duty context, incorporate objective or repeated sleep and fatigue measures, and test whether the observed associations persist over time.

## Figures and Tables

**Table 1:** Latent-class model-selection indices.

Classes	Log Likelihood	Parameters	AIC	BIC	CAIC	SABIC	Entropy	Smallest Modal Class, %
1	−4104.5	9	8227.1	8272.6	8281.6	8244.0	1.000	100.0
2	−3970.5	19	7979.0	8075.0	8094.0	8014.6	0.690	35.7
3	−3930.9	29	7919.8	8066.3	8095.3	7974.2	0.749	21.2
4	−3920.1	39	7918.3	8115.3	8154.3	7991.4	0.751	13.4
5	−3913.3	49	7924.6	8172.2	8221.2	8016.5	0.706	5.8

AIC, Akaike information criterion; BIC, Bayesian information criterion; CAIC, consistent AIC; SABIC, sample-size-adjusted BIC. BIC was the primary enumeration criterion.

**Table 2:** Participant characteristics by modal latent-class assignment.

Characteristic	Lower Frequency	Intermediate Frequency	Higher Frequency
Modal class size, n (%)	563 (48.7)	347 (30.0)	245 (21.2)
Estimated class prevalence, %	47.7	28.3	24.0
Average posterior probability	0.896	0.788	0.988
Age, years	31.99 ± 9.46	26.80 ± 5.98	25.98 ± 5.37
Female, %	11.4	4.3	2.4
Troops, %	65.9	87.6	89.8
BMI, kg/m^2^	25.16 ± 2.75	25.31 ± 2.58	25.21 ± 2.59
Weekly work, h	44.16 ± 12.67	43.47 ± 12.18	44.60 ± 13.25
Weekly training, min	355.8 ± 165.4	394.6 ± 207.5	436.4 ± 230.5
Daily smoking, %	11.9	21.6	31.0
Sleep duration, h	6.18 ± 1.08	6.20 ± 1.08	5.85 ± 1.25
Sleep efficiency, %	87.96 ± 10.97	86.70 ± 11.70	85.25 ± 12.53
Physical fatigue, 0–10	6.31 ± 1.89	6.56 ± 2.01	6.74 ± 1.89

Values are mean ± standard deviation unless otherwise stated. Modal assignment is presented only to describe the observed composition of the classes; inferential outcome models propagated posterior classification uncertainty. BMI, body mass index.

**Table 3:** Adjusted associations between latent beverage-frequency profile and sleep and fatigue outcomes.

Outcome	Contrast	n	Adjusted Difference	95% CI	*p*	BH q
Sleep duration, h	Intermediate vs. lower	1128	0.018	−0.158 to 0.194	0.843	—
Sleep duration, h	Higher vs. lower	1128	−0.230	−0.416 to −0.045	0.015	0.022
Sleep efficiency, percentage points	Intermediate vs. lower	998	−1.239	−3.200 to 0.721	0.215	—
Sleep efficiency, percentage points	Higher vs. lower	998	−2.129	−4.177 to −0.082	0.041	0.041
Physical fatigue, 0–10	Intermediate vs. lower	1022	0.198	−0.137 to 0.532	0.246	—
Physical fatigue, 0–10	Higher vs. lower	1022	0.395	0.077 to 0.714	0.015	0.022

Models adjusted for age, sex, body mass index, military-rank group, weekly work hours, weekly training volume, perceived excessive workload, cohort, water-intake score, alcohol-intake score, and smoking status. Estimates were pooled across 300 posterior pseudo-class draws using Rubin’s rules. Benjamini–Hochberg q values apply to the prespecified higher-versus-lower family across the three outcomes; intermediate-versus-lower contrasts were secondary.

## Data Availability

The participant-level dataset is not publicly available because it contains information from active-duty military personnel and is subject to ethical and institutional restrictions. De-identified data required to verify the reported analyses may be considered by the corresponding author upon reasonable request and subject to approval by the relevant ethics and military authorities. The analysis code is provided as Supplementary Materials.

## Supplementary Materials

The following supporting information can be downloaded at: https://www.mdpi.com/article/10.3390/nu18172930/s1, Figure S1. Bayesian information criterion across candidate models. Table S1. Latent-class model-selection indices. Table S2. Conditional response probabilities for the selected three-class model. Table S3. Secondary intermediate-versus-lower class contrasts. Table S4. Sensitivity analyses. Table S5. Mutually adjusted individual beverage indicators. Table S6. Larger-cohort model-enumeration sensitivity.

## Abbreviations

AIC, Akaike information criterion; BH, Benjamini–Hochberg; BIC, Bayesian information criterion; BMI, body mass index; CAIC, consistent Akaike information criterion; CI, confidence interval; LCA, latent class analysis; SABIC, sample-size-adjusted Bayesian information criterion.
