# Supplementary material for: Beverage Co-Consumption Profiles, Sleep, and Fatigue in Professional Army Personnel
Source: Nutrients. 2026 Sep 7;18(17):2930. doi: 10.3390/nu18172930 (PMC13566972; doi:10.3390/nu18172930)
Supplement: Supplementary file 1 [file nutrients-18-02930-s001.zip › nutrients-4486626-supplementary.pdf]

Supplementary Materials

Beverage Co-Consumption Profiles, Sleep, and Fatigue in Army Personnel

Table S1. Latent-class model-selection indices

Classes	Log likelihood	Parameters	AIC	BIC	CAIC	SABIC	Entropy	Smallest class, %
1	-4104.5	9	8227.1	8272.6	8281.6	8244.0	1.000	100.0
2	-3970.5	19	7979.0	8075.0	8094.0	8014.6	0.690	35.7
3	-3930.9	29	7919.8	8066.3	8095.3	7974.2	0.749	21.2
4	-3920.1	39	7918.3	8115.3	8154.3	7991.4	0.751	13.4
5	-3913.3	49	7924.6	8172.2	8221.2	8016.5	0.710	5.8

AIC, Akaike information criterion; BIC, Bayesian information criterion; CAIC, consistent AIC; SABIC, sample-size-adjusted BIC. BIC was the primary enumeration criterion.

Table S2. Conditional response probabilities for the selected three-class model

Item and category	Lower	Intermediate	Higher
Caffeine: Never	0.253	0.084	0.000
Caffeine: 1–2/day	0.555	0.781	0.747
Caffeine: 3–4/day	0.169	0.104	0.191
Caffeine: ≥5/day	0.023	0.031	0.062
Sugar-sweetened drinks: Rarely/never	0.609	0.240	0.209
Sugar-sweetened drinks: 1–2/week	0.267	0.561	0.276
Sugar-sweetened drinks: 3–4/week	0.080	0.184	0.264
Sugar-sweetened drinks: Almost daily	0.044	0.016	0.251
Energy drinks: Never	0.571	0.000	0.000
Energy drinks: Occasionally	0.429	0.381	0.048
Energy drinks: 1–2/week	0.000	0.619	0.103
Energy drinks: ≥3/week	0.000	0.000	0.849

Figure S1. Bayesian information criterion across candidate models

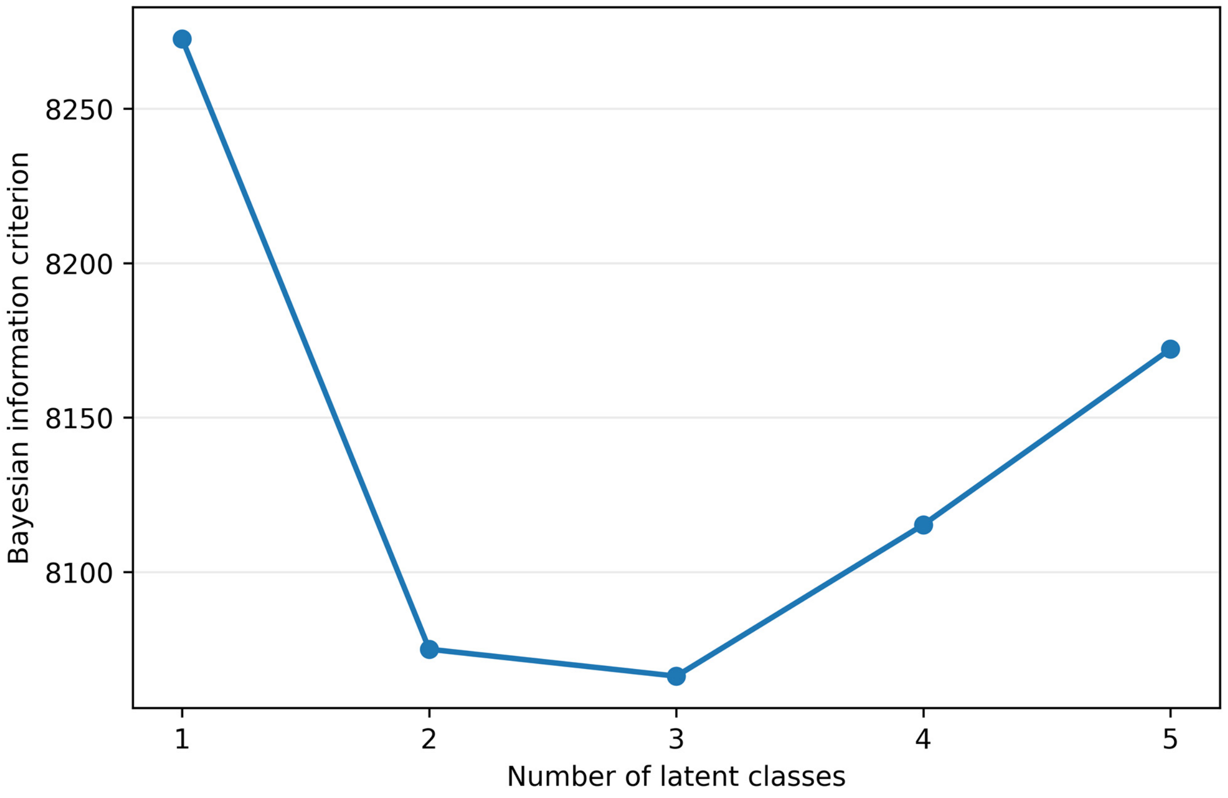

The three-class model minimized BIC.

Table S3. Secondary intermediate-versus-lower class contrasts

Outcome	n	Estimate	SE	95% CI	p
Sleep duration, h	1128	0.018	0.090	-0.158 to 0.194	0.843
Sleep efficiency, percentage points	998	-1.239	1.000	-3.200 to 0.721	0.215
Physical fatigue, 0–10	1022	0.198	0.170	-0.137 to 0.532	0.246

Table S4. Sensitivity analyses

Analysis	Outcome	n	Estimate	95% CI	p
Ordered class trend	Sleep duration	1128	-0.106	-0.197 to -0.014	0.023
Ordered class trend	Sleep efficiency	998	-1.078	-2.081 to -0.074	0.035
Ordered class trend	Physical fatigue	1022	0.198	0.038 to 0.357	0.015
Modal class + unit fixed effects	Sleep duration	1128	-0.112	-0.201 to -0.022	0.014
Modal class + unit fixed effects	Sleep efficiency	998	-1.182	-2.161 to -0.203	0.018
Modal class + unit fixed effects	Physical fatigue	1022	0.236	0.082 to 0.390	0.003
Continuous score, per SD	Sleep duration	1128	-0.111	-0.178 to -0.043	0.001

Analysis	Outcome	n	Estimate	95% CI	p
Continuous score, per SD	Sleep efficiency	998	-0.902	-1.704 to -0.101	0.027
Continuous score, per SD	Physical fatigue	1022	0.198	0.077 to 0.318	0.001
Continuous score + sleep/psychological covariates	Physical fatigue	1022	0.195	0.074 to 0.317	0.002

Table S5. Mutually adjusted individual beverage indicators

Outcome	Beverage indicator	n	Estimate per SD	95% CI	p
Sleep duration	Caffeine	1128	-0.019	-0.090 to 0.052	0.603
Sleep duration	Sugar-sweetened drinks	1128	-0.072	-0.147 to 0.003	0.059
Sleep duration	Energy drinks	1128	-0.061	-0.142 to 0.019	0.135
Sleep efficiency	Caffeine	998	-0.070	-0.854 to 0.714	0.861
Sleep efficiency	Sugar-sweetened drinks	998	-0.440	-1.329 to 0.449	0.332
Sleep efficiency	Energy drinks	998	-0.701	-1.537 to 0.135	0.100
Physical fatigue	Caffeine	1022	0.119	-0.022 to 0.260	0.097
Physical fatigue	Sugar-sweetened drinks	1022	0.056	-0.072 to 0.183	0.392
Physical fatigue	Energy drinks	1022	0.111	-0.029 to 0.252	0.121

Table S6. Larger-cohort model-enumeration sensitivity

Classes	AIC	BIC	Entropy	Smallest modal class, %
1	7428.5	7473.0	1.000	100.0
2	7196.5	7290.5	0.694	40.9
3	7131.8	7275.3	0.800	21.5
4	7134.8	7327.8	0.781	12.8

The larger cohort contained 1,043 participants. The expected caffeine, sugar-sweetened drink, and energy-drink scores for the three classes were 0.953/0.542/0.435, 1.088/1.003/1.614, and 1.324/1.530/2.946, respectively.

Additional Analytical Notes

The selected three-class model was re-estimated from 100 random starts using a separate stability check. The best log likelihood was −3930.8822; 45 starts converged within 0.01 log-likelihood units of this value. Bivariate residual statistics for caffeine × sugar-sweetened drinks, caffeine × energy drinks, and sugar-sweetened drinks × energy drinks were 14.75 ($p = 0.098$), 2.59 ($p = 0.978$), and 4.64 ($p = 0.864$), respectively.
